# Asymmetries and hemispheric interaction in the auditory system of elderly people

**DOI:** 10.3389/fnimg.2023.1320989

**Published:** 2024-01-03

**Authors:** Nicole Angenstein

**Affiliations:** Combinatorial NeuroImaging Core Facility, Leibniz Institute for Neurobiology, Magdeburg, Germany

**Keywords:** aging, auditory cortex, hemispheric specialization, lateralization, neuroimaging

## Abstract

Age-related changes of asymmetries in the auditory system and decreasing efficiency of hemispheric interaction have been discussed for some time. This mini-review discusses recent neuroimaging studies on alterations in lateralization of cortical processing and structural changes concerning the division of labor and interaction between hemispheres during auditory processing in elderly people with the focus on people without severe hearing loss. Several changes of asymmetries in anatomy, function and neurotransmitter concentration were observed in auditory cortical areas of older compared to younger adults. It was shown that connections between left and right auditory cortex are reduced during aging. Functionally, aging seems to lead to a reduction in asymmetry of auditory processing. However, the results do not always point into the same direction. Furthermore, correlations between function, anatomy and behavior in the left and right hemisphere appear to differ between younger and older adults. The changes in auditory cortex asymmetries with aging might be due to compensation of declining processing capacities, but at the same time these mechanisms could impair the balanced division of labor between the two hemispheres that is required for the processing of complex auditory stimuli such as speech. Neuroimaging studies are essential to follow the slow changes with aging as in the beginning no behavioral effects might be visible due to compensation. Future studies should control well for peripheral hearing loss and cognitive decline. Furthermore, for the interpretability of results it is necessary to use specific tasks with well-controlled task difficulty.

## Introduction

Older adults often have difficulties in understanding speech especially during noisy conditions (Glyde et al., 2011). Age-related hearing difficulties are present also despite normal peripheral hearing (Füllgrabe et al., 2014). In addition to age-related peripheral hearing loss and cognitive decline, central auditory processing changes with age, e.g., the division of labor between hemispheres and the efficiency of hemispheric interaction (Martin and Jerger, 2005; Bellis and Jorgensen, 2013). An efficient division of labor and interaction between hemispheres, however, is required for processing complex auditory stimuli because different acoustic features are processed differently in the left and right hemisphere. For example, the left hemisphere is specialized for temporal processing and the right hemisphere for spectral processing (Zatorre and Belin, 2001). For processing of complex auditory stimuli such as speech and music, the simultaneous processing of different acoustic parameters is essential. Changes in lateralization and impaired hemispheric interaction with aging might lead to a decline in central auditory processing in addition to age-related peripheral hearing loss and cognitive decline. Understanding the mechanisms of decline in central auditory processing is very important, as hearing difficulties are one of the main risk factors for developing dementia (Livingston et al., 2020).

There are two main models for the effect of aging on hemispheric asymmetries (Ocklenburg and Güntürkün, 2018). The right hemi-aging model is supported by behavioral studies and assumes that right hemispheric functions (e.g., visuo-spatial abilities) are more strongly impaired by aging than left hemispheric functions (e.g., verbal abilities) (Dolcos et al., 2002). The HAROLD (hemispheric asymmetry reduction in older adults) model was derived in the context of asymmetries in episodic memory retrieval and is mainly supported by neuroimaging studies. It assumes that prefrontal activity is less lateralized in older than younger adults (Cabeza, 2002). The author suggested that more bilateral activity in older adults might have a compensatory function (counteract cognitive decline) or might reflect a dedifferentiation process (difficulties to recruit specialized neuronal processes). However, these two models are not specific for alterations in lateralization of processing in the auditory domain. The mini-review discusses recent neuroimaging studies on alterations in the lateralization of cortical processing and structural changes concerning the division of labor and interaction between hemispheres during auditory processing in elderly people with the focus on people without severe hearing loss (Table 1).

**Table 1:** Overview of cited original studies with age range, number of participants (*N*), hearing status, used methods and tasks.

**Study**	**Age (years)**	***N***	**Hearing**	**Methods/tasks**
Berlingeri et al. (2013)	26.5 ± 4.4 62 ± 7.6	24 24		fMRI Sentence judgement task, sentence recognition task
Chen et al. (2013)	18–27 55–75	10 8	Average hearing threshold ≤ 25 dB at 125 Hz−4 kHz	Spectroscopy
Dias et al. (2020)	19–30 56–82	22 25	≤ 40 dB HL at 250 Hz−4 kHz; interaural asymmetry ≤ 15 dB	DTI ^*^ITD digit segregation
Dobri and Ross (2021)	19–29 69–90	21 22	Self-reported normal hearing	Spectroscopy
Dobri et al. (2022)	19–28 69–87	19 19		Spectroscopy
Eddins et al. (2022)	24.9 ± 2.5 70.0 ± 2.7	10 10	Younger: ≤ 25 dB HL at 250 Hz−8 kHz; older: ≤ 25 dB HL at 250 Hz−4 kHz, ≤ 60 dB above 4 kHz	EEG Passive listening
Edgar et al. (2014)	37.90 ± 10.88	29		Structural MRI MEG, ASSR
Edgar et al. (2017)	39.6 ± 12.1	53		Structural MRI MEG, EEG, ASSR
Farahani et al. (2020)	20–30 50–60 70–80	19 20 16	≤ 25 dB HL at 125 Hz−4 kHz	EEG ASSR
Fuksa et al. (2022)	26 ± 0.7 32 ± 2.6 60 ± 4.1 65 ± 1.8 72 ± 2.1 68 ± 2.1	15 15 12 12 12 12	Normal hearing + tinnitus Mild presbycusis + tinnitus Expressed presbycusis +tinnitus	fMRI Words, words in babble, modulated pink noise, babble
Giroud et al. (2018)	20–29 67–84	13 23	≤ 30 dB at 500 Hz−4 kHz; interaural asymmetry ≤ 15 dB	Resting state EEG Structural MRI ^*^frequency selectivity, ^*^temporal compression, ^*^speech in noise
Giroud et al. (2019)	20–29 67–84	15 23	≤ 30 dB at 500 Hz−4 kHz; interaural asymmetry ≤ 15 dB	Structural MRI EEG, MMN ^*^fundamental frequency discrimination
Goossens et al. (2016)	20–30 50–60 70–80	19 20 14	≤ 25 dB HL at 125 Hz−4 kHz	EEG, ASSR
Isler et al. (2021)	19–29 67–84	27 24	≤ 30 dB HL at 500 Hz−4 kHz; interaural asymmetry ≤ 15 dB	EEG, MMN Structural MRI ^*^vowel discrimination
Keller et al. (2019)	70.64 ± 4.66	26	≤ 30 dB HL at 500 Hz−2 kHz; interaural asymmetry ≤ 14 dB	fMRI, structural MRI Pattern-matching task
Liem et al. (2014)	25 ± 3	23		fMRI, structural MRI pattern-matching task
Liu et al. (2022)	21.7 ± 1.7 66.4 ± 3.2	22 21		fMRI, structural MRI Semantic task in contrast to tone discrimination
Manan et al. (2013)	23–37 41–67	29 24	Self-reported normal hearing	fMRI Listening to noise Working memory task in quiet and noise
Nenert et al. (2017)	18–76	224		fMRI Semantic decision task Covert verb generation
Plessen et al. (2014)	7–59	215		Structural MRI
Profant et al. (2014)	24.34 ± 0.51 67.9 ± 0.45 70.38 ± 1.18	20 17 17	Normal hearing Mild presbycusis Expressed presbycusis No significant differences between ears	Structural MRI, DTI
Profant et al. (2015)	22–30 65–72 64–79	18 15 15	≤ 20 dB at 125 Hz−12.5 kHz ≤ 20 dB at 125 Hz−4 kHz Exceed physiological range starting at 1 kHz	fMRI Indicating presence of noise
Stadler et al. (2023)	18–38 57–73	20 17	≤ 20 dB at 125 Hz−4 kHz Mixed normal hearing and presbycusis	Structural MRI, DTI fMRI Categorization and comparison of direction of frequency modulations

In some studies, tasks were not performed during the functional measurement (marked with ^*^).

## Anatomical alterations

Diffusion tensor imaging (DTI) data suggest a decline in connections between hemispheres via the corpus callosum with aging based on measured reduction in fractional anisotropy (FA) and increase in mean diffusivity (MD) (Ota et al., 2006; Lebel et al., 2010). These alterations suggest a decrease in myelination and reduced number of axons in older compared to younger adults (Tromp, 2016). FA is also specifically reduced in fibers connecting the auditory cortices of both hemispheres (Stadler et al., 2023). This decline in interhemispheric anatomical connections in older adults might affect the functional division of labor between hemispheres because hemispheric interaction depends on its advantages and costs (Banich, 1998).

Altered corpus callosum integrity can correlate with altered performance: younger and older adults with lower callosal FA showed lower performance in spatial digit segregation (Dias et al., 2020). The older adults showed lower callosal FA and lower task performance than the younger adults. From their analysis, the authors suggested that age-related deficits in spatial digit segregation are explainable by individual callosal FA values.

In addition to alterations of anatomical connections between hemispheres with aging, asymmetric atrophy in left and right hemispheric regions were observed. Plessen et al. (2014) described a stronger reduction of cortical thickness among other regions in right temporal regions with age in people aged 7–59 years. With an activation likelihood estimation meta-analysis, Minkova et al. (2017) observed converging evidence for a right-lateralized gray matter loss in the superior temporal gyrus (STG). Whereas in other areas a left-lateralization of gray matter loss was found (supramarginal gyrus, inferior parietal lobe, postcentral gyrus). In the same line, Stadler et al. (2023) showed stronger right hemispheric reduction of auditory cortex (AC) regions-of-interests (ROIs) in older adults derived from probability maps (Amunts et al., 2020, 2021). In the temporal lobe, the results of these studies fit to the right hemi-aging model (Dolcos et al., 2002).

Profant et al. (2014) did not observe that age-related atrophy changed the left-lateralization of gray matter volume and gyral surface area of Heschl's gyrus (HG) or planum temporale (PT) in participants with mild and expressed presbycusis. However, from their DTI data they suggested that the white matter under the left HG is stronger affected by age than under the right HG (trend for stronger decrease in MD and axial vector of diffusion in the white matter under left HG). From their data, they also suggested that changes in gray and white matter are affected predominately by age and not by presbycusis.

Different correlations between atrophy in AC areas and behavior between younger and older adults were also observed. By investigating the PT, older adults showed bilateral lower cortical volume, thickness and surface area (Isler et al., 2021). In older adults, these values correlated positively with performance in vowel discrimination meaning that higher atrophy was associated with lower performance. In younger adults, lower cortical thickness in left PT was associated with better performance which was interpreted as a possible sign of efficient neural organization. Overall, the differences in correlation were interpreted to suggest that speech processing becomes more bilateral with aging.

Other studies observed that cortical thickness was lower in normal-hearing older than in younger adults in both left and right auditory areas, whereas cortical surface area did not differ between younger and older adults (Giroud et al., 2018, 2019). However, only in older adults, thicker right PT was associated with better frequency selectivity performance and thicker right Heschl's sulcus (HS) with better temporal compression performance (Giroud et al., 2018). Also only in older adults, bigger cortical surface area in the right HG and right STG were associated with better frequency selectivity performance. In addition, older people with larger cortical thickness in the right HS showed better performance in speech in noise perception. This is in line with their interpretation that older adults rely more on prosodic cues, the processing of which is known to be right lateralized. Similarly, in older adults, better performance in fundamental frequency discrimination was related to larger cortical thickness in the right STG (Giroud et al., 2019). In contrast, a positive correlation between mismatch-negativity (MMN) on stress within a word and cortical surface area of left STG and left HS were observed. Their interpretation was that in older adults with larger surface in left auditory areas, the perception of complex spectro-temporal pattern might be more robust against aging effects. However, a test on the correlation in the group of younger adults was not reported in this study.

## Functional alterations—fMRI studies

With two auditory and two visual tasks, Berlingeri et al. (2013) observed that the HAROLD effect is not only present in the prefrontal cortex. Older adults showed a reduction of the left-lateralized processing observed in younger adults during a sentence judgement task and a sentence recognition task (a long-term memory task) in language processing areas (STG, inferior frontal gyrus). The authors suggested that the HAROLD model might be a specific form of the compensation-related utilization of neural circuits (CRUNCH) hypothesis that suggests that older adults recruit additional resources for compensation to increase performance (Reuter-Lorenz and Cappell, 2008).

In another study, older adults did not show the rightward lateralization for the processing of suprasegmental speech cues that was present in younger adults (Liem et al., 2014; Keller et al., 2019). In younger adults, the lateralization in the investigated auditory regions HG, PT, posterior STG were shifted to the right hemisphere when the locally time-reversed segments of sentences got longer (Liem et al., 2014), whereas this was not the case in older adults (Keller et al., 2019). In addition, links between functional lateralization and behavioral task performance and anatomy were observed in the older adults (Keller et al., 2019): older adults whose lateralization of processing in the PT and posterior STG changed less with the variation of stimulation (length of time-reversed segments) performed better in the pattern-matching task. In addition, older adults with higher cortical thickness in the right PT showed less variability in functional lateralization across different time-reversed segment lengths.

A study with a high amount of left-handers (77 out of 224) from 18 to 76 years suggested that the reduction of language lateralization with age is strongly influenced by handedness and sex (Nenert et al., 2017). A decrease of the lateralization index with age was observed only in male right-handers and only in the temporo-parietal ROI during covert verb generation on acoustically presented nouns and not in a semantic decision task. However, the authors used masks that spanned multiple functional areas and thus these masks were possibly too unspecific to identify age-related effects on lateralization that may be present only in small areas.

In a study with a semantic task in comparison to a tone discrimination task, older adults showed the same overall activation pattern and lateralization as younger adults after controlling for gray matter percentage, age and reaction time during tone discrimination (Liu et al., 2022). However, older adults showed weaker functional interhemispheric connectivity between right middle frontal gyrus and left STG than younger adults. Furthermore, while there was no correlation between performance and brain responses in the younger group, better performance in the older group was accompanied by greater left-lateralization of activity in the supramarginal gyrus and stronger interhemispheric fronto-parietal connections.

In another study, processing in younger and older adults were compared with two tasks that differently require hemispheric interaction (Stadler et al., 2023). Older adults stronger involved AC areas, especially during the task that strongly requires hemispheric interaction, i.e., comparison of direction of frequency modulations (Brechmann et al., 2007; Angenstein and Brechmann, 2013). Furthermore, especially during this task, left and right AC activity correlated with activity in several areas intra- and interhemispherically, e.g., areas for language processing, working memory, attention and motor planning. However, during both tasks the correlation between left and right AC activity was higher in younger than older adults. This was explained by lower anatomical connections in older than in younger participants.

During indicating the appearance of pink noise, older adults with both mild and expressed presbycusis showed stronger right lateralized processing than younger adults (Profant et al., 2015). Similarly, during hearing babble noise and word working memory tasks, older adults showed a more rightward shift of asymmetry compared to the left-lateralized asymmetry in the STG and HG of younger adults (Manan et al., 2013).

A mixed group of older adults with mild and expressed presbycusis with and without tinnitus showed stronger activity in the left AC and less lateralization of activity compared to younger adults with a combined analysis of different acoustic stimulation (words, words in babble, modulated pink noise and babble) (Fuksa et al., 2022). However, it is not clear how much this result was affected by tinnitus and presbycusis.

## Functional alterations—EEG and MEG studies

Effects of age on resting-state EEG power were investigated in two frequency bands relevant for speech processing (theta for prosody, intonation and low gamma for formant transitions, voice onset times) at auditory ROI electrodes (Giroud et al., 2018). Theta power showed rightward asymmetry in older but not in younger adults. Theta power for the younger group and gamma power for both groups did not show a significant lateralization. However, gamma and theta power showed a trend for more right lateralization in the older compared to the younger group. Furthermore, beside the above-mentioned relation between anatomy and performance, they also observed a correlation between lateralization of resting state theta power and anatomy in older adults. Only in older adults, thicker cortex around the right superior temporal sulcus predicted stronger right-lateralization of theta power. This also fits to their suggestion that older adults rely more on prosodic cues during speech processing than younger adults.

In another study, older adults showed reduced contralateral lateralization of processing during passive listening to noise bursts with different interaural time differences (ITD), although the overall responses were larger in older adults (Eddins et al., 2022). This reduction was more pronounced for right-leading stimuli suggesting a hemispheric difference in the reduction of contralaterality with aging.

With source analyses on auditory steady-state responses (ASSR), Farahani et al. (2020) observed for different frequencies of amplitude modulations (4, 20, 40, 80 Hz) and depending on the side of stimulation different effects of age on lateralization. For 80 Hz modulation, the right-lateralized asymmetry of activity in subcortical structures observed in younger adults was absent in older adults with exceptionally good hearing. For AC sources, reduction of lateralization, no change or even reversal of lateralization compared to younger adults were observed. The results showed that there are alterations in hemispheric asymmetry in temporal processing with aging. Further interpretations are difficult because even in younger adults the lateralization of ASSR for AC sources varied strongly with frequency and side of stimulation and was not always consistent with previous studies. Sensor level analysis showed mainly a reduction of ASSR asymmetry in older adults for 80 Hz amplitude modulations (Goossens et al., 2016). For 40 Hz ASSR, reduced total power and inter-trial coherence were observed with aging in the left but not right hemisphere (Edgar et al., 2014, 2017).

## Metabolic alterations

With magnetic resonance spectroscopy, a higher GABA level in the left than in the right AC was detected in younger but not in older adults (Dobri and Ross, 2021; Dobri et al., 2022). The authors suggested that this reduction in GABA asymmetry might be an explanation for age-related deficits in auditory hemispheric interaction (Martin and Jerger, 2005). Furthermore, they observed a correlation between GABA level in the right AC of older adults and age and performance in a speech-in-noise test, i.e., lower GABA level correlated with higher age and lower performance (Dobri and Ross, 2021). Their interpretation was that lower GABA level leads to lower temporal precision, making feature binding more difficult, which in turn complicates separation of speech from noise. In addition to differences in metabolite concentrations in the left and right AC without stimulation, differences were also detected after pure tone stimulation: younger adults showed a strong right-sided asymmetry of (glutamine/glutamate)/creatine concentration and a left sided asymmetry for GABA/creatine which were both strongly reduced in older adults (Chen et al., 2013). The authors interpreted this by a disappearance of hemispheric dominance for tone processing in older adults.

## Discussion

Recent neuroimaging studies not only confirmed previous behavioral and electrophysiological studies (Martin and Jerger, 2005; Bellis and Jorgensen, 2013) but also provided new insights in asymmetry differences and alterations in hemispheric interaction in the auditory system in older compared to younger adults. Asymmetric atrophy was observed, however, not in all studies. Furthermore, several anatomical measures in left and right auditory cortical areas differently correlate with behavior in older compared to younger adults. Asymmetries of neurotransmitter concentrations are reduced in older adults. Connections between left and right AC decrease during aging. Functionally, aging seems to lead to a reduction in asymmetry of auditory processing. However, the results do not always point in the same direction. The functional changes in AC asymmetries during aging might be due to compensation of decreasing processing capacities, but at the same time these mechanisms could impair the well-balanced functional division of labor between hemispheres. Neuroimaging studies are essential to follow these slowly developing processes with aging as in the beginning no behavioral effects might be visible due to compensation, whereas compensatory processes can be observed through increased activity, e.g., shown by the contralateral noise procedure (Angenstein and Brechmann, 2013, 2015, 2017; Brechmann and Angenstein, 2019; Stadler et al., 2023).

Inconsistent results of the individual studies might be due to different age ranges and different hearing thresholds of participants. For example, it has been shown in older adults that hearing impairment led to stronger decline in brain volume in right than left temporal regions compared to normal-hearing people (Lin et al., 2014). Furthermore, tasks, passive listening or only the detection of the presence of a stimulus were used. Using detection tasks or passive listening might reduce effects of general cognitive decline on results. However, in these cases the specificity of the results for auditory processing is reduced. In addition, there are large methodological differences between studies, e.g., using sensor- or source-level analyses with the same EEG-data can already lead to different results regarding the lateralization of processing (Goossens et al., 2016; Edgar et al., 2017; Farahani et al., 2020). Sometimes an apparent reduction of lateralization due to aging might simply be the result of missing activity in older adults (Berlingeri et al., 2013).

Future studies need to decipher further the effects of changes in functional and anatomical hemispheric asymmetries and reduced hemispheric interaction with age, age-related hearing loss and declining cognitive abilities, all of which influence each other in various ways (Humes et al., 2012; Rönnberg et al., 2013; Cardin, 2016; Ebaid and Crewther, 2020; Uchida et al., 2021; Lentz et al., 2022). Therefore, all studies should be conducted with young control participants who are comparable in terms of their peripheral hearing and cognitive abilities. For the interpretability of the results, specific tasks with well-controlled difficulty should always be used, since task difficulty influences hemispheric involvement in addition to the given tasks (Brechmann and Angenstein, 2019), and age effects are more pronounced with complex stimuli or demanding tasks (Martin and Jerger, 2005; Bellis and Jorgensen, 2013). For DTI measurements, the use of MRI metrics that are robust against crossing fibers (Figley et al., 2021) might reveal additional age-related hemispheric differences in the integrity of the auditory pathway.

Answering the open questions will help to develop diagnostic methods and treatment for age-related hearing impairment that are caused at least partly by changes in the division of labor between hemispheres with aging. Possible interventions should strengthen interhemispheric connectivity or adjust lateralization of auditory processing, e.g., by music-based intervention (Quinci et al., 2022), listing training with contralateral noise (Brechmann and Angenstein, 2019; Stadler et al., 2023) or transcranial stimulation.

## Author contributions

NA: Conceptualization, Writing – original draft, Writing – review & editing.
